# Recent Advances in κ-Carbide Precipitation Behavior and Its Influence on Mechanical Properties in Austenite-Based Fe-Mn-Al-C Lightweight Steels

**DOI:** 10.3390/ma19040727

**Published:** 2026-02-13

**Authors:** Yanjie Mou, Kai Lei, Jiahao Li, Xiaofei Guo, Jianwen Fan, Chundong Hu, Han Dong

**Affiliations:** 1 State Key Laboratory of Materials for Advanced Nuclear Energy, Shanghai 200444, China; myj_1994@shu.edu.cn (Y.M.); leikaisd@shu.edu.cn (K.L.); lijiahao1223@foxmail.com (J.L.); huchundong99@163.com (C.H.); 2 Zhejiang Institute of Advanced Materials, Shanghai University, Jiaxing 314100, China; 3 School of Materials Science and Engineering, Shanghai University, Shanghai 200444, China; 4 AT&M Environmental Engineering Technology Co., Ltd. (AEET), No. 76, Xueyuan Nanlu, Haidian, Beijing 100081, China

**Keywords:** Fe-Mn-Al-C steels, austenite, κ-carbides, dislocation, precipitation strengthening

## Abstract

Austenitic Fe-Mn-Al-C lightweight steels have attracted considerable interest for automotive applications due to their exceptional specific strength, where κ-carbides precipitation critically influences mechanical properties. This review systematically examines the crystal structure, classification, and precipitation kinetics of κ-carbides, emphasizing their spatial distribution-dependent effects: coarse κ-carbides at austenite grain boundaries induce embrittlement and degrade toughness, while nanoscale κ’-carbides within grains enhance strength and ductility through dislocation interactions (e.g., Orowan bypassing and shearing), activating deformation mechanisms such as Dynamic Slip Band Refinement (DSBR), Shear Band-Induced Plasticity (SIP), and Microband-Induced Plasticity (MBIP). Thermodynamic calculations guide alloy design to ensure a single-phase austenite structure at the typical hot-rolling finishing temperature (~900 °C), avoiding harmful phases while promoting beneficial precipitates. Mn suppresses κ-carbide formation, whereas Al and C act as promoters, with intragranular κ’-carbides favoring higher Al/C concentrations (e.g., >6.2% Al and >1.0% C). Heat treatment parameters critically influence κ-carbide distribution, where rapid cooling (e.g., water quenching) suppresses κ-carbides, and subsequent aging (500–700 °C) enables homogeneous precipitation of κ’-carbides. Pre-deformation prior to annealing further accelerates κ-carbide nucleation by introducing crystal defects. Optimal performance requires integrated composition-processing-microstructure optimization to achieve a nnanoscaleκ’-carbide-strengthened austenite matrix through controlled composition and thermo-mechanical processing to achieve an optimal strength-ductility balance.

## 1. Introduction

With escalating resource depletion and environmental concerns, energy conservation and emission reduction have become global priorities. As indispensable transportation tools, automobiles exert immense pressure on resources and the environment. According to the International Organization of Motor Vehicle Manufacturers (OICA), global vehicle ownership reached 1.5 billion in 2024. This magnitude underscores the critical need for automotive lightweighting to reduce emissions.

Lightweight automotive steels achieve energy savings by enhancing strength and reducing density (high specific strength). Current research focuses on Fe-Mn-Al-C alloy steels, classified into four categories based on microstructure: ferritic steels, ferrite-based duplex steels, austenite-based duplex steels, and austenitic steels. The most promising are austenite-based duplex and austenitic steels (collectively termed austenite-based alloy steels). High Mn, Al, and C contents in these steels form diverse microstructures and precipitates (e.g., B2, DO_3_, κ-carbides) [1,2,3,4,5,6]. The B2 phase (ordered body-centered cubic, CsCl-type) and DO_3_ phase (ordered face-centered cubic, FeAl_3_-type) are intermetallic compounds primarily based on Fe-Al(-Mn) ordering. These precipitates, along with κ-carbides, significantly influence strength and plasticity. Among these, the influence of the κ-phase on the strengthening mechanisms of austenite-based alloy steels is the most remarkable [1,7]. It affects not only dislocation motion but also acts as a hydrogen trap, thereby improving the resistance to hydrogen embrittlement in these steels [8]. As a result, the κ-phase plays a dominant role in determining the mechanical properties of the material. Consequently, research on precipitates in austenite-based alloy steels has primarily focused on κ-carbides.

This paper synthesizes data from numerous studies to analyze the effects of alloying elements, rolling, and heat treatment on κ-carbide precipitation kinetics. It further examines the role of κ-carbides in micro-deformation theories during plastic deformation, establishing principles for the composition design and processing of austenite-based alloy steels.

## 2. Overview of κ-Carbides

**Figure 1:**
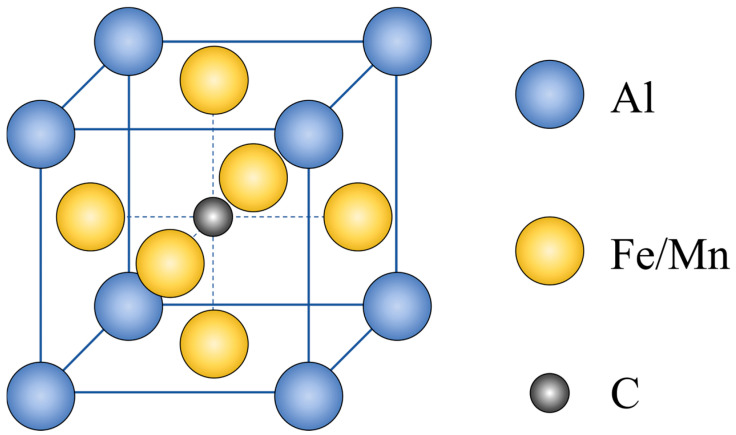
Schematic illustrations of unit cell structures of κ-carbides.

κ-carbides (κ-phase) precipitate in austenite-based steels when C and Al exceed threshold levels. Figure 1 shows the unit cell structure of κ-phase. Ideally, Al atoms occupy the 8 corner positions, Fe/Mn atoms occupy the 6 face-centered positions, and C atoms reside at the body-centered position [9].

### 2.1. Classification of κ-Carbides

**Figure 2:**
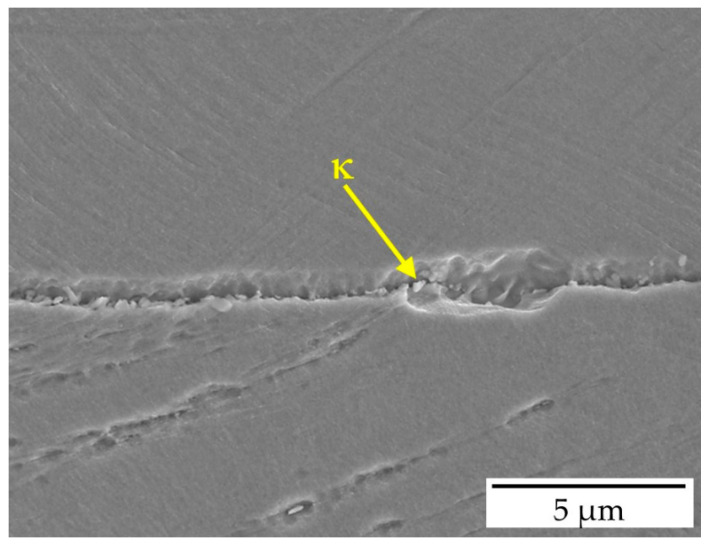
κ-carbides distributed at grain boundaries [17].

Based on precipitation sites, κ-carbides are classified into two types [10,11,12,13]: intergranular κ*-carbides at austenite grain boundaries and intragranular κ’-carbides within austenite grains. This spatial distinction is critical as it governs their stability and morphology. κ*-carbides form at higher temperatures where faster diffusion kinetics promote heterogeneous nucleation at γ/γ interfaces, initially precipitating as discrete coarse particles with coherent or semi-coherent interfaces to at least one adjacent grain (Figure 2) [14,15,16,17]. With prolonged aging, κ*-carbides grow into adjacent grains, developing lamellar morphologies [18].

**Figure 3:**
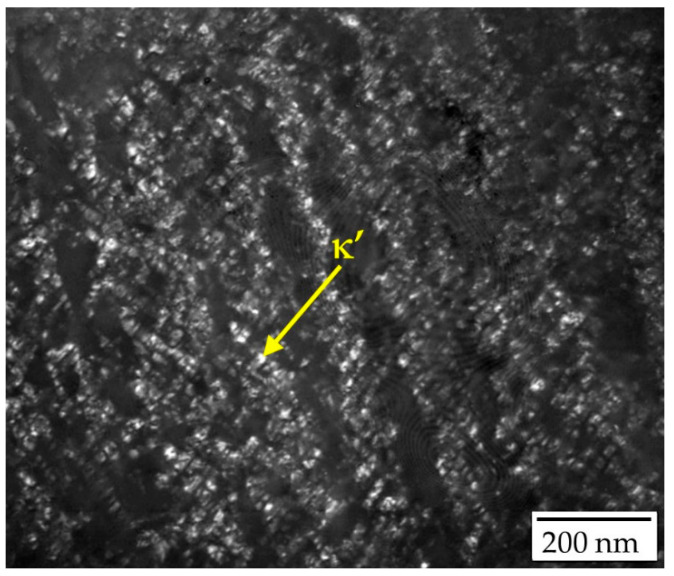
TEM image showing the distribution of κ′-carbides within austenite grains [17].

**Figure 4:**
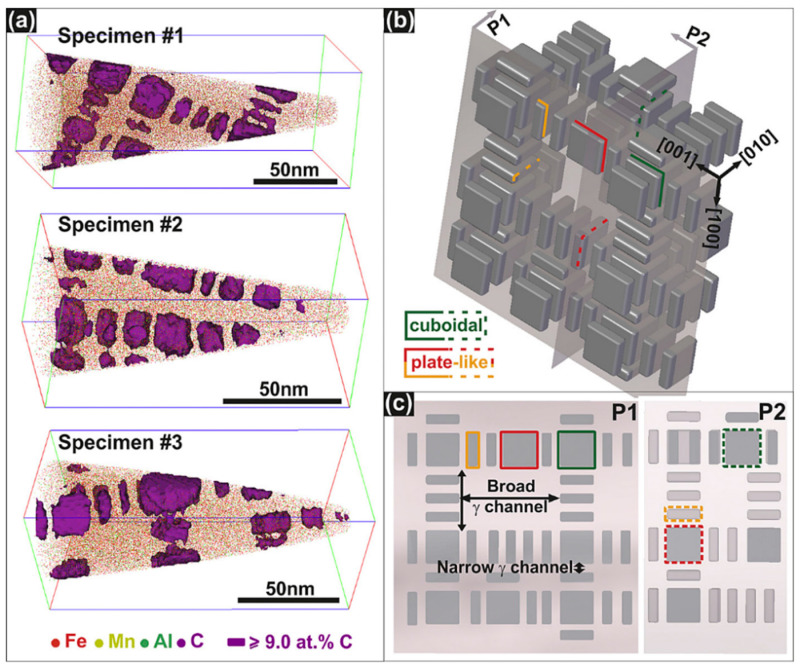
(**a**) Reconstructed three-dimensional APT maps of Fe (red), Mn (yellow), Al (green), and C (purple). κ-carbide precipitates are shown at the 9% C iso-concentration surface; (**b**) Schematic diagram of three-dimensional morphology and arrangement of carbide precipitates based on APT observation; (**c**) Two-dimensional projection of κ/γ microstructure along the <001> direction in (**b**), reflecting TEM observations [21].

κ’-carbides precipitate intragranularly during cooling or aging as metastable, fine, and uniformly distributed particles. In some alloy systems, κ’-carbides can also precipitate within B2-ordered [19] or α-ferrite [20] phase domains. Compared to κ*-carbides, κ′-carbides are finer and more uniform (Figure 3). Yao et al. [21] used atom probe tomography (APT) to map the 3D morphology and 2D projection along <001> of κ′-carbides in Fe-30.4Mn-8Al-1.2C steel aged at 600 °C for 24h (Figure 4). The γ-matrix channels between κ′-carbides are uneven, with wide channels (10–40 nm) and narrow channels (2–5 nm).

### 2.2. Precipitation Behavior of κ-Carbides

It is generally accepted that, under sufficient diffusion kinetics (e.g., slow cooling or isothermal aging), austenite decomposition proceeds through solute partitioning and redistribution. High-temperature austenite decomposes into solute-lean γ′ and solute-rich γ″ phases. Further cooling triggers short-range ordering (SRO) in γ″, transforming it into the L1_2_ phase. Continued SRO allows C atoms to migrate to L1_2_ body centers, forming κ′ [22,23,24,25,26]. The sequence is: γ → γ′ + γ″ → γ′ + L1_2_(SRO) → γ′ + κ′.

Recent studies [27], however, suggest κ-carbides nucleate directly from disordered γ-phase without solute partitioning, indicating unresolved formation mechanisms. Chu et al. [28] proposed κ-phase exists post-quenching, with aging facilitating growth. C diffusion governs growth, while Mn/Al exhibits limited long-range diffusion. Emo et al. [29] modeled lattice sites using FM (Fe/Mn), Al, vacancies (V), and C. Site occupancy factors ($f_{s}^{FM}$, $f_{s}^{\mathrm{Al}}$, $f_{s}^{V}$,$f_{i}^{C}$) range from 0 to 1, with $f_{s}^{FM}+f_{s}^{Al}+f_{s}^{V}=1$. System free energy (*G*) is as follows:

(1) $$G=H^{s}+H^{i}-H^{pe}+T\left(S^{s}+S^{i}\right)$$

where $H^{s}$, $H^{i}$ are substitutional/interstitial enthalpies, $H^{pe}$ is pure-element enthalpy, and $S^{s}$, $S^{i}$ are entropies.

Cooling media also alter κ-carbide characteristics. Zhang et al. [30] showed slower cooling (brine → water → oil → air) increases κ-carbide volume fraction and size, enhancing hardness but reducing impact toughness. Water cooling maximizes impact energy, while air cooling promotes κ*-carbides, degrading toughness. Lu et al. [26] noted that cooling rate influences κ-matrix interface properties, affecting formability.

Thus, κ-carbide precipitation kinetics are governed by composition, heat treatment, and cooling conditions. Optimizing these parameters tailors κ-carbide behavior, enhancing Fe-Mn-Al-C steel performance.

### 2.3. Influence of κ-Carbides on Strengthening Mechanisms

κ-carbides primarily strengthen austenitic steels via precipitation hardening. Given that microstructures are predominantly austenitic (containing <30% δ-ferrite and minor carbides), the mechanical properties are governed by austenite deformation behavior. Stacking fault energy (SFE) critically determines deformation mechanisms [31,32,33,34,35,36,37,38,39,40,41,42,43,44,45,46]: at low SFE, transformation-induced plasticity (TRIP) dominates; medium SFE facilitates twinning-induced plasticity (TWIP); while high SFE promotes dislocation slip as the primary mechanism [47,48,49,50]. Elevated SFE suppresses both TRIP and TWIP effects but enhances dislocation glide. However, dislocation slip demonstrates lower efficiency in contributing to plasticity compared to TRIP or TWIP. Aluminum significantly increases SFE, and when Al content exceeds 7 wt.%, TWIP becomes fully inhibited [1].

κ-carbides precipitate preferentially at these high aluminum concentrations, where dislocation slip governs deformation. Therefore, investigating interactions between κ-carbides and dislocations is essential to elucidate their strengthening role.

κ*-carbides coarsen at grain boundaries, embrittling interfaces and degrading ductility [51,52,53,54]. They are detrimental and must be avoided via composition/processing design.

κ′-carbides enhance mechanical properties by interacting with dislocations [3,4,21,26,30,32,33,34,52,55,56,57,58,59,60,61,62,63,64]. Under stress, dislocations glide and are impeded by κ′-carbides. Higher κ′ volume fraction/size increases dislocation-obstacle interactions, elevating yield strength. Yao [21] identified two interaction mechanisms: Orowan looping and particle shearing, governed by anti-phase boundary energy (γ_APB_) and interparticle spacing. Orowan looping dominates at γ_APB_ > 700 mJ/m^2^ and spacing > 20 nm. Orowan strengthening ($\sigma_{Oro}$) is [65] as follows:

(2) $$\sigma_{Oro}=\left(\frac{10.8\cdot f^{1/2}}{X}\right)\cdot \left(ln\left(\frac{X}{6.125\times 10^{-4}}\right)\right)$$

where *f* = volume fraction, *X* = average diameter.

Wide γ-channels (10–40 nm) permit Orowan looping. Narrow channels (2–5 nm) prevent loop formation, forcing dislocations to shear κ′-carbides.

Flow stress ($\sigma_{tot}$) in Fe-Mn-Al-C steels is [44] as follows:

(3) $$\sigma_{tot}=\sigma_{F}+\sigma_{SS}+\sigma_{GB}+\sigma_{P}+\sigma_{SRO}+\sigma_{SH(\varepsilon )}$$

where $\sigma_{F}$ = Peierls-Nabarro friction, $\sigma_{SS}$ = solid solution strengthening, $\sigma_{GB}$ = Hall-Petch grain boundary strengthening, $\sigma_{P}$ = precipitate strengthening, $\sigma_{SRO}$ = short-range order strengthening, $\sigma_{SH(\varepsilon )}$= strain hardening. Summing non-strain-dependent terms gives $\sigma_{0}$ (yield strength), simplifying to [44] as follows:

(4) $$\sigma_{tot}=\sigma_{0}+\sigma_{SH(\varepsilon )}$$

The flow behavior of these materials is significantly influenced by the precipitation of κ-carbides under varying deformation temperatures and strain rates [66]. It is well documented that the plastic deformation mechanisms in austenitic lightweight steels primarily include Dynamic Slip Band Refinement (DSBR) [21,44], Shear Band-Induced Plasticity (SIP) [12], and Microband-Induced Plasticity (MBIP) [32,33].

#### 2.3.1. Dynamic Slip Band Refinement (DSBR)

Welsch et al. [44] proposed the Dynamic Slip Band Refinement (DSBR) mechanism during their investigation of deformation mechanisms in Fe-30.4Mn-8Al-1.2C steel. When plastic deformation initiates under applied stress, dislocation sources activate in the form of Frank-Read sources and grain boundary sources to emit dislocations. As dislocations interact with each other and with short-range ordered (SRO) phases, additional dislocation sources form loops that expand and detach from their sources via cross-slip associated with the established Frank-Read bowing mechanism. The expansion of dislocation loops destroys any compositional order (including both SRO and long-range order, LRO) on their slip planes, thereby inducing slip plane softening that facilitates the generation of new dislocation loops from the same source.

Consequently, activated Frank-Read sources can readily emit large numbers of dislocations on identical slip planes. The emitted dislocations ultimately reach grain boundaries or become trapped within the grain by dislocations on parallel slip bands. Due to the pronounced order-induced planar slip behavior, the generated dislocations rarely leave the slip planes, resulting in complete dislocation filling of the slip planes. The accumulated dislocations generate back stress acting on dislocation sources, which increases with the number of emitted dislocations. Once the local stress at the source falls below the critical activation stress, the source ceases to emit further dislocation loops. Under these conditions, slip planes become saturated with dislocations that manifest as distinct slip bands traversing the grain.

It should be specifically noted that while initial slip plane softening promotes slip band formation, the increasing dislocation density within the bands ultimately leads to slip plane hardening due to the aforementioned back stress. This hardening causes source exhaustion, halting the evolution of individual slip bands. With increasing applied stress, other dislocation sources within the microstructure activate and generate new slip bands. These newly formed slip bands repeat the described evolutionary process, progressively reducing inter-band spacing. This cyclic mechanism constitutes the core of the Dynamic Slip Band Refinement (DSBR) process. Through DSBR, the quenched Fe-30.4Mn-8Al-1.2C steel achieves an ultimate tensile strength of 1440 MPa with 46% elongation.

Given that κ′-carbides are exclusively aligned along ⟨001⟩ directions while face-centered cubic (FCC) slip occurs on {111}<110> systems, dislocations encounter κ′-carbide aggregates with narrow γ-channels immediately ahead—despite the presence of wide γ-channels elsewhere in the matrix. Consequently, dislocation passage through κ′-carbides predominantly occurs via shearing rather than Orowan looping. The contribution of Orowan strengthening is therefore negligible, and the strain hardening stress ($\sigma_{SH(\varepsilon )}$) arising from DSBR can be expressed as [44] follows:

(5) $$\sigma_{SH(\varepsilon )}=K\cdot M\cdot G\cdot b/D$$

where *K* is the geometric factor, *M* = 3.06 denotes the Taylor factor for randomly oriented grains, *G* = 70 GPa represents the shear modulus of the alloy, *b* = 0.26 nm is the magnitude of the Burgers vector, and *D* signifies the average slip band spacing.

**Figure 5:**
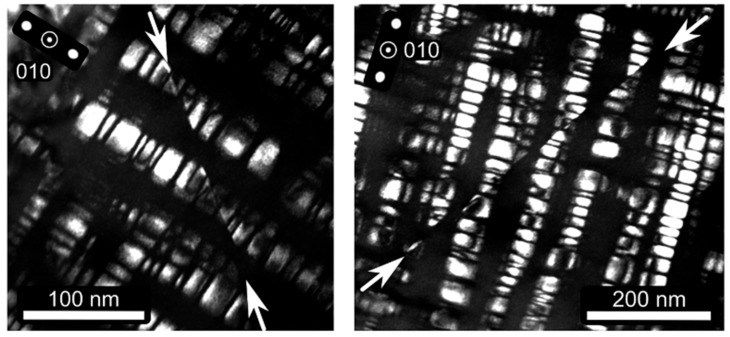
TEM observation of κ′-carbide morphology and dislocation-mediated shearing of κ′-carbides (indicated by white arrows) during early-stage deformation at a strain of 0.02 [21].

In their investigation of Dynamic Slip Band Refinement behavior in Fe-30.4Mn-8Al-1.2C steel, Yao et al. [21] directly observed the shearing of κ′-carbides by dislocations during plastic deformation, as indicated by the arrows in Figure 5.

#### 2.3.2. Shear Band-Induced Plasticity (SIP)

**Figure 6:**
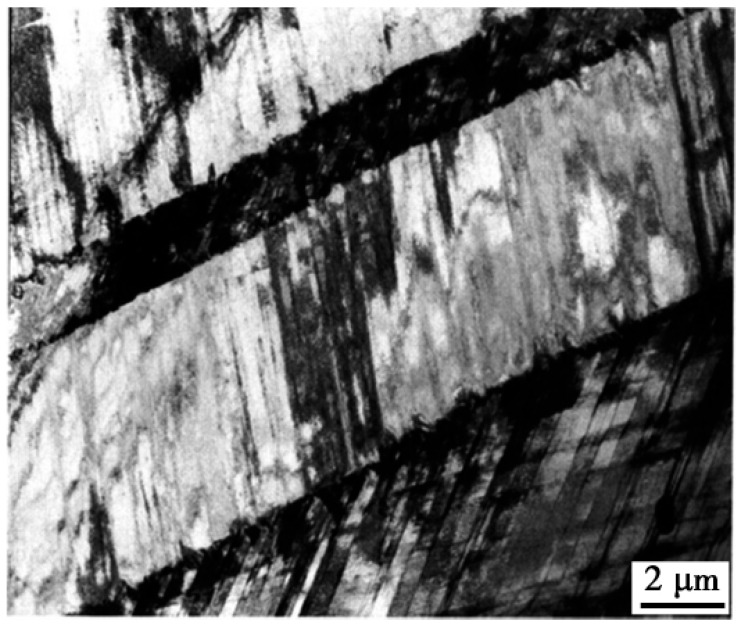
TEM bright-field image showing shear bands on the {111} planes in the austenite matrix [12].

Frommeyer et al. [12] investigated austenite-based Fe-(26–28)Mn-(10–12)Al-(1.0–1.2)C alloy steels comprising an austenitic matrix, δ-ferrite (6–8%), and nano-scaled κ′-carbides. The homogeneous dispersion of κ′-carbides within the alloy microstructure facilitated the formation of numerous uniformly distributed shear bands within austenite grains during plastic deformation, as evidenced by TEM analysis in Figure 6. Frommeyer et al. attributed the superior tensile strength (875 MPa) and elongation (58%) of Fe-(26–28)Mn-(10–12)Al-(1.0–1.2)C steels to this homogeneous shear deformation of austenite. This phenomenon, characterized by extensive uniform shear bands enabling homogeneous plastic deformation in austenite, is designated as Shear Band-Induced Plasticity (SIP).

Complementary research by Moon et al. [65] demonstrated that varying Mo content (0–3 wt.%) in Fe-30Mn-10.5Al-1.1C alloys effectively modulates κ′-carbide precipitation. Their results revealed an inverse correlation between Mo content and κ′-carbide characteristics: increasing Mo additions reduced both the volume fraction and size of κ′-carbides. This microstructural evolution fundamentally altered deformation mechanisms—SIP dominated at higher κ′-carbide concentrations, while Microband-Induced Plasticity (MBIP) prevailed at lower precipitation densities. Consequently, incremental Mo additions (up to 3 wt.%) enhanced yield strength and ductility while maintaining virtually unchanged ultimate tensile strength. 

#### 2.3.3. Microband-Induced Plasticity (MBIP)

In their systematic investigation of Fe-28Mn-9Al-0.8C and Fe-28Mn-10Al-1.0C alloy steels, Yoo et al. [32,33] established that both steels exhibit a fully austenitic microstructure following solution treatment and quenching processes, with short-range ordering (SRO) domains of characteristic dimensions less than 2 nm present within the austenite matrix. Microstructural analysis revealed that at 5% applied strain, the obstruction of dislocations by SRO domains leads to the formation of parallel dislocation arrays within austenite grains. With progressive strain increase, the sequential activation of additional slip systems results in a continuous reduction in inter-slip plane spacing, ultimately leading to the development of well-defined Taylor lattices (TLs). Further strain accumulation induces distinct TL rotation, subsequently facilitating the formation of domain boundaries (DBs) and the evolution of microbands (MBs).

**Figure 7:**
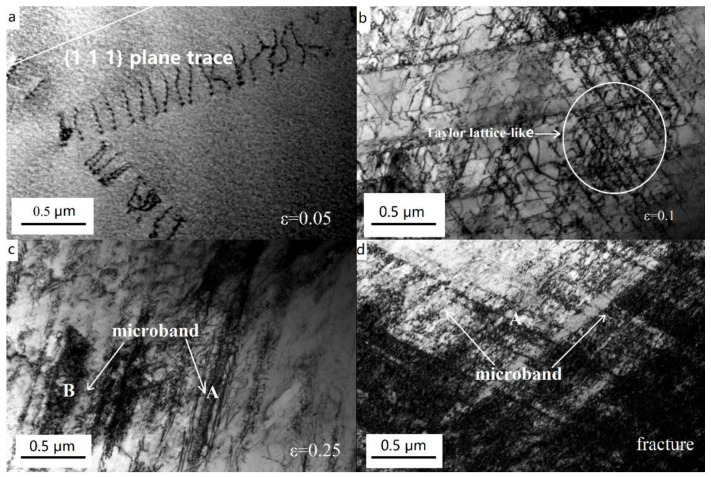
TEM micrographs of low-density steel after being interrupted at different strains during room temperature tensile deformation of (**a**) 5%, (**b**) 10%, (**c**) 25%, and (**d**) failure to fracture [67].

Similarly, Ma et al. [67] conducted comparable research. They systematically characterized the dislocation configurations in the steel via interrupted tensile tests at different strain levels (0.05, 0.15, 0.25) and after final fracture, as shown in Figure 7. Their study revealed that at a low strain of 5%, the substructure development was characterized by dislocation pile-ups on a single slip plane and slip along the {111} plane, exhibiting typical planar slip configurations (Figure 7a). When the strain increased to 10%, the dislocation structure remained planar, but slip traces on another plane were observed, indicating the activation of multiple slip systems and the formation of a Taylor lattice-like structure, a type of low-energy dislocation configuration (Figure 7b). Upon further straining to 25%, microbands with distinct boundaries were observed (marked ‘A’ in Figure 7c). With increasing strain, the spacing between slip bands decreased, and a pronounced microband structure developed (marked ‘B’ in Figure 7c). At fracture, the slip traces became finer and more intensive, and the substructure was dominated by intersections of microbands. Notably, the dislocation structure maintained a planar slip character throughout deformation, and no dislocation cells were formed, even in the fractured sample (Figure 7d). This work provides further insight into the plastic deformation mechanism of this steel from a dislocation evolution perspective.

The study demonstrated that microband formation and intersection simultaneously enhance both strength and plasticity, a phenomenon designated as Microband-Induced Plasticity (MBIP). The MBIP mechanism typically operates at higher strain levels and may coexist with Dynamic Slip Band Refinement (DSBR). Crucially, the strain hardening contribution described by Equation (5) for DSBR remains equally applicable to MBIP-dominated deformation [68].

However, Microband-Induced Plasticity (MBIP), Dynamic Slip Band Refinement (DSBR), and Shear Band-Induced Plasticity (SIP) cannot fully account for the work-hardening behavior of age-hardened austenite-based alloy steels; further in-depth investigations into the interactions between κ′-carbides and dislocations are required.

### 2.4. Influences of κ-Carbides on Mechanical Properties

The mechanical performance of austenitic Fe-Mn-Al-C lightweight steels is critically governed by the spatial distribution of κ-carbides, exhibiting a strength-toughness tradeoff. This dichotomy originates from the interactions of intergranular κ*-carbides and intragranular κ’-carbides with dislocations and the resultant crack initiation.

#### 2.4.1. Embrittlement Mechanism of Intergranular κ*-Carbides

Intergranular κ*-carbides, characterized by their coarse and frequently continuous morphology along austenite grain boundaries, act as stress concentration sites. Their brittle nature and interfacial incoherence facilitate microcrack nucleation and provide low-energy paths for intergranular fracture, leading to a pronounced deterioration in impact toughness and ductility. Kim et al. [51] reported that intergranular κ-carbides in Fe-20Mn-11.5Al-1.2C steel significantly reduce ductility by promoting crack initiation along grain boundaries. This embrittlement effect is markedly exacerbated under conditions with the coarsening of κ-carbides, such as slow cooling rates or prolonged isothermal aging. Xiong et al. [8] reported both a distinct reduction in ductility and hydrogen embrittlement resistance in a Fe-30Mn-8Al-1.2C steel with intergranular κ-carbides at prolonged aging at 600 °C. Consequently, microstructural design strategies suggest suppressing κ-carbide formation through rapid quenching from the austenitizing temperature, as demonstrated by Zhang et al. [30], who showed that water quenching minimizes κ-carbide formation and preserves toughness in Fe-Mn-Al-C steels or micro-alloying with elements like Ni or Mo to minimize κ-carbides at grain boundaries, as evidenced by Moon et al. [65] for Mo addition and Kim et al. [69] for Ni addition.

#### 2.4.2. Strengthening and Strain Hardening Mediated by Intragranular κ’-Carbides

In contrast, homogeneous dispersion of nanoscale intragranular κ’-carbides provides effective strengthening while concurrently raising strain hardening. These precipitates impede dislocation glide through two primary mechanisms: Orowan looping in wider matrix channels and particle shearing in narrower ones, with the operative mechanism dictated by the anti-phase boundary energy (γ_APB) and interparticle spacing [21,44]. Shearing of ordered κ’-carbides disrupts the local L12 superlattice structure, inducing pronounced planar slip behavior [21,44]. This localized deformation mode activates enhanced strain hardening, including Dynamic Slip Band Refinement (DSBR) [44], Shear Band-Induced Plasticity (SIP) [12], and Microband-Induced Plasticity (MBIP) [32,33], which collectively facilitate a superior synergy between tensile strength and uniform elongation. Rahnama et al. [5] reported that optimally aged Fe-Mn-Al-C steel exhibited a product of strength and elongation exceeding 40 GPa·%, significantly outperforming conventional TWIP steels. Yao et al. [21] further confirmed that κ’-carbide size below 15 nm promotes the shearing mechanism, maximizing strain hardening capacity.

Optimal mechanical performance requires maximizing strengthening by raised volume fraction of κ’-carbides through controlled aging (500–700 °C) while suppressing the embrittling κ*-carbides via tailored composition design and processing routes [30,65].

## 3. Alloying Effects on κ-Carbide Precipitation Kinetics

### 3.1. The Influences of the Mn Element

Manganese (Mn) exerts a suppressive effect on κ-carbide formation [70,71], though it is not the dominant influencing factor [1]. In steel, the primary role of Mn is to stabilize austenite. In austenite-based Fe-Mn-Al-C alloy steels, the Mn content inherently exceeds 5 wt.% (typically >25 wt.%) to stabilize the austenitic matrix. Consequently, Mn’s impact on κ-carbide precipitation kinetics can be considered secondary in alloy design considerations for this system.

Notably, tensile properties remain relatively consistent within the Mn concentration range of 22–30 wt.%. However, exceeding 30 wt.% Mn triggers the precipitation of β-Mn phase—a complex cubic (P4132) intermetallic compound known to induce severe embrittlement through grain boundary segregation and interfacial decohesion. This precipitation-driven embrittlement mechanism necessitates a strict upper limit of 30 wt.% Mn in compositional design protocols.

### 3.2. The Influences of Al and C Elements

Aluminum (Al) and carbon (C) exert significant influences on the precipitation kinetics of κ-carbides. Based on thermodynamic calculations performed using the FactSage^®^ software, Chen et al. [1] systematically investigated the effect of Al content on the phase equilibria of Fe–Mn–Al–C alloy steels under conditions of fixed Mn content (20 wt.%) and C content (0.85 wt.%). The thermodynamic results reveal a clear alloying trend: with increasing Al content, the formation temperature of the κ phase increases markedly, indicating an enhancement in its thermodynamic stability. When the Al content reaches approximately 4.5 wt.%, κ-carbides begin to precipitate at around 600 °C. Further increases in Al content substantially broaden the temperature range over which the κ phase is stable. At an Al content of about 7 wt.%, the κ phase can remain thermodynamically stable down to temperatures as low as approximately 200 °C.

**Figure 8:**
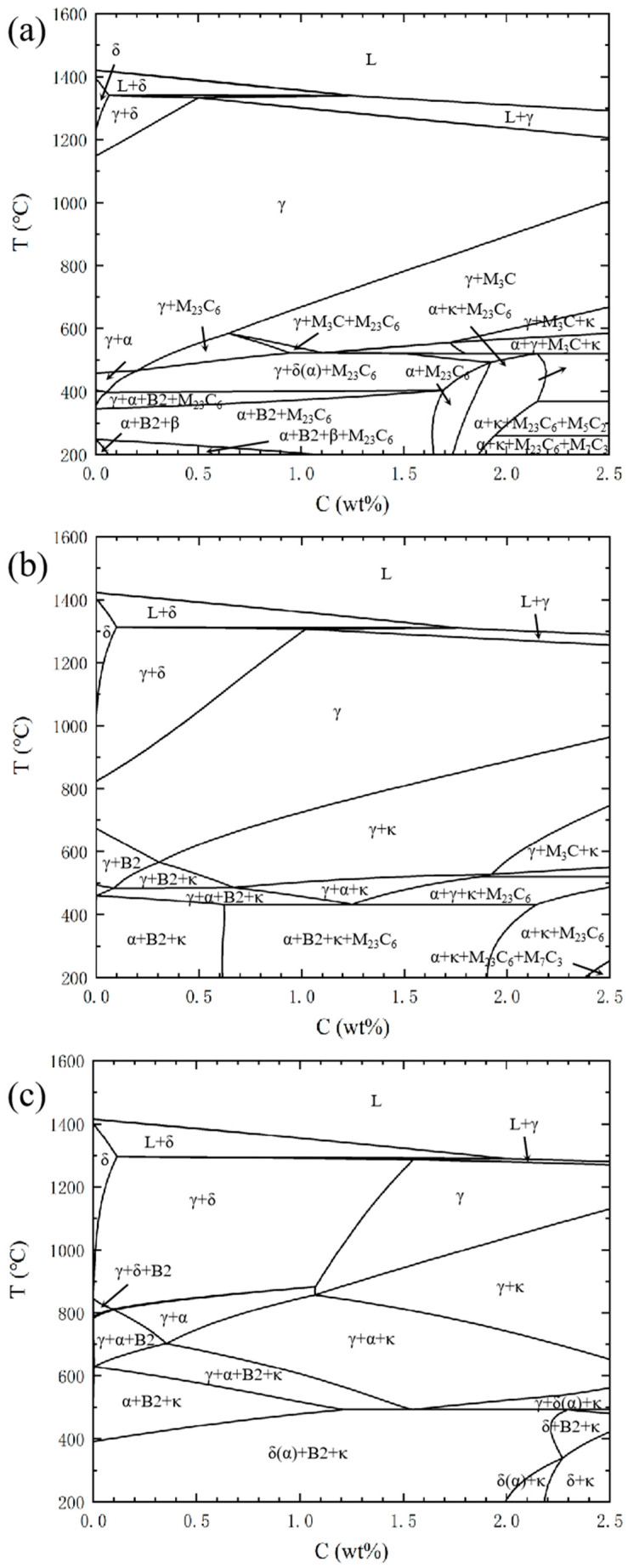
Phase diagrams of Fe-Mn-Al-C alloy steel calculated at an Mn content of 30% and Al content of (**a**) 4%, (**b**) 7%, and (**c**) 10% using Thermo-Calc software.

Figure 8 presents the computationally derived phase diagrams for Fe-Mn-Al-C alloy steels at 30 wt.% Mn with varying aluminum contents (4, 7, and 10 wt.% Al), generated using Thermo-Calc thermodynamic software. Computational analysis reveals a pronounced aluminum-dependent phase evolution: increasing Al content progressively contracts the γ-austenite single-phase region while simultaneously expanding both the δ-ferrite single-phase field and γ + δ dual-phase domain. This systematic phase redistribution demonstrates aluminum’s critical role in thermodynamically stabilizing δ-ferrite within this alloy system. Concurrently, the computational results establish that irrespective of aluminum concentration, incremental carbon additions systematically expand the temperature range of κ-carbide stability. This carbon-dependent phase field extension manifests most significantly at elevated temperatures (>600 °C), confirming carbon’s parallel function in enhancing κ-phase stability through preferential carbide formation energetics.

The precipitation behavior of κ-carbides exhibits significant sensitivity to aluminum and carbon concentrations. Ding et al. [72] systematically demonstrated that increasing carbon content substantially enhances κ-phase precipitation volume fraction, establishing a direct positive correlation between carbon content and precipitation kinetics. Complementary research by Song et al. [48] combined first-principles calculations with in situ synchrotron X-ray diffraction to reveal that κ-phase ordering achieves exceptional thermodynamic stability within high-aluminum austenitic matrices, providing fundamental insights into κ-phase formation energetics. Huang’s [73] comprehensive study on Fe-(23-31)Mn-(2-10)Al-(0.4-1.0)C steels solution-treated to single-phase austenite identified critical precipitation thresholds during isothermal aging at 650 °C: intragranular κ′-carbides required >6.2 wt.% Al and >1.0 wt.% C, while intergranular κ*-carbides initiated at >5.5 wt.% Al and >0.7 wt.% C. Alloys below these compositional limits showed no κ-phase formation even after 360 h of aging.

Contemporary research by Peng et al. [74] employed machine learning algorithms to establish composition-microstructure relationships in low-density Fe-Mn-Al-C steels, predicting and experimentally verifying in Fe-30Mn-8Al-1C steel that κ-phase stability could be maintained with aluminum concentrations as low as ~4 wt.% at 30 wt.% Mn. This confirms consistent elemental influence trends across alloy systems, though minimum concentration thresholds exhibit composition-dependent variability due to complex multi-component interactions.

Elevating aluminum and carbon concentrations synergistically promotes κ-carbide precipitation through dual mechanisms: enhanced chemical driving force for precipitation and increased austenite lattice parameter (Δaγ = +0.00094 nm per wt.% Al). The lattice expansion reduces coherency strain during κ′-carbide nucleation, significantly facilitating intragranular precipitation. To quantify this relationship, Huang et al. [73] established a composition boundary expression (in wt.%) for the precipitation of the intragranular κ′ phase in Fe-Mn-Al-C alloys based on the critical austenite lattice constant (aγ,crit) for the formation of intragranular κ′-carbides:

0.098(wt.%Al) + 0.208(wt.%C) = 1 − 0.0054(wt.%Mn)(6)

The critical lattice constant of the austenite matrix is approximately 0.3670 nm. This parameter fundamentally influences the coherency strain energy during κ′-carbide precipitation, with lattice mismatch below 1.5% being thermodynamically favorable for intragranular precipitation.

The industrial-scale manufacturability of austenite-based alloy steels fundamentally depends on their phase stability at industrial hot-rolling temperatures, which in modern production lines are typically around 900 °C. Thus, compositional design at 900 °C is of critical importance. Chen et al. [1] systematically investigated the phase equilibria of Fe-(10, 20, 30)Mn-xAl-yC alloys at 900 °C by integrating experimental data [75,76] with CALPHAD thermodynamic calculations [77]. Their study clearly indicates that to avoid harmful intergranular precipitation during thermomechanical processing, alloy compositions must be strictly designed to lie within the γ single-phase region. The key to this design is precise control of carbon content to completely eliminate δ-ferrite while maintaining a stable single-phase γ microstructure. This optimization strategy effectively suppresses the formation of brittle phases and promotes uniform plastic deformation within the austenite matrix, thereby ensuring optimal ductility performance of the material.

### 3.3. The Influences of Other Elements

Contemporary alloy design research increasingly focuses on trace element additions to modulate κ-carbide behavior. Kim et al. [69] demonstrated that nickel (Ni) additions effectively refine intragranular κ′-carbide dimensions while simultaneously contributing to solid solution strengthening. Notably, Ni suppresses κ-carbide precipitation by enhancing γ-phase stability through reduced carbon activity. Complementary research by Kies et al. [2] revealed that strategic Ni-Co co-additions in Fe-26.9Mn-14.6Al-4.9C high-Mn steels promote κ-carbide precipitation while concurrently forming B2-phase precipitates, resulting in synergistic strengthening.

Conversely, silicon (Si) additions exhibit contrasting effects. Wang et al. [78] established that Si enhances the thermodynamic activity of both aluminum and carbon in Fe-30Mn-9Al-1.2C steel, thereby accelerating κ-carbide nucleation kinetics. In contrast, transition metals like molybdenum (Mo) and chromium (Cr) exert inhibitory effects. Moon et al. [65,79,80,81] systematically documented that Mo/Cr additions impede κ-carbide formation through solute drag at precipitation interfaces. This suppression mechanism was quantitatively validated by Bai et al. [82], who reported a critical threshold of >5 wt.% Cr is causing a significant reduction in κ-phase precipitation density due to inhibited nucleation thermodynamics.

Xie et al. [83] observed that 0.5 wt.% V additions simultaneously enhance κ-phase precipitation and ferrite formation through partitioned elemental segregation. More significantly, Yang et al. [84] demonstrated that 3–5 wt.% Cu not only promotes κ-carbide precipitation but also reduces ferrite content and alters its morphology. After heat treatment at 500 °C for 20 h, substantial co-precipitation of nanoscale Cu-rich particles and κ-carbides occurred in Cu-containing steels, where Cu-rich particle formation promoted subsequent κ-carbide precipitation. This synergistic precipitation mechanism resulted in a remarkable increase in yield strength by approximately 289 MPa in the 5 wt.% Cu steel while maintaining ductility. This exceptional strength-ductility balance is attributed to nanoscale precipitate distribution and improved matrix-precipitate coherency, offering new pathways for designing high-performance lightweight steels with optimized strength-ductility synergy.

**Figure 9:**
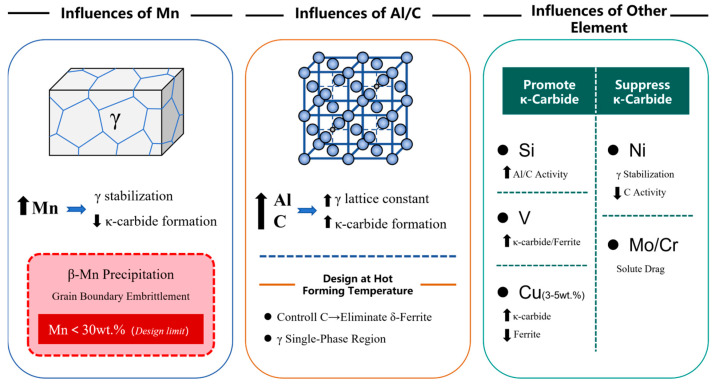
Summary of the impact of the main chemical composition on κ-carbides precipitation in austenite-based lightweight steels.

In summary, to optimize the performance of the κ-phase, the compositional design of austenite-based lightweight steels was proposed in Figure 9.

## 4. The Influences of Processing Technology on the Kinetics of κ-Phase Precipitation

Processing parameters significantly influence the precipitation kinetics of κ-carbides through thermally activated mechanisms, with temperature playing a critical governing role in nucleation and growth behavior. The thermomechanical processing sequence—comprising hot-rolling temperature, solution treatment parameters, cooling rate, and aging conditions—exerts multi-level control over the precipitation process. Specifically, the thermal history during hot rolling and solution treatment determines austenite stability and solute supersaturation, directly modulating the nucleation density of the κ-phase. Subsequent cooling rates govern diffusion-dominated growth kinetics. Accelerated quenching can effectively suppress the formation of coarse intergranular κ*-carbides, and optimized quenching processes [63,83,85,86,87,88,89] can entirely inhibit such intergranular precipitation while simultaneously promoting a fine dispersion of intragranular κ′-carbides. Isothermal aging within the 500–700 °C window enables precise control over precipitate coarsening behavior. Crucially, as discussed in Section 3.2 regarding compositional design principles, hot-rolling and solution treatment temperatures must ensure the alloy composition resides within the γ single-phase region at 900 °C to prevent detrimental phase transformations during industrial processing.

**Figure 10:**
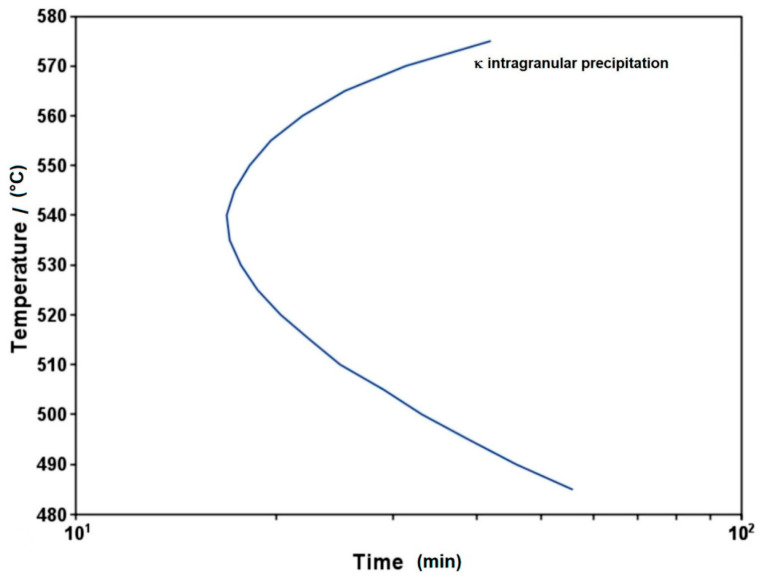
TC-PRISMA-calculated TTP diagram for the κ phase [90].

The Time-Temperature-Precipitation (TTP) diagram for intragranular κ-phase precipitation in Fe-30Mn-8.8Al-0.3Si-0.15C steel is presented in Figure 10. The precipitation process encompasses three stages: nucleation, growth, and coarsening. Lopez-Hirata et al. [90] determined the interfacial free energy between the precipitate and the matrix to be approximately 0.083 J·m^−2^, corresponding to a coherent interface. According to the “nose” of the C-curve, the intragranular κ-phase exhibits the fastest growth kinetics near 540 °C. The precipitate size increases from an initial size of approximately 2 nm to about 20 nm after aging at 550 °C for 200 h, with the process transitioning into a coarsening-dominated stage after approximately 50 h of aging.

The precipitation of κ/κ′ carbides is recognized as a core strengthening mechanism in Fe–Mn–Al–C lightweight steels. Research by Zhou et al. [63] shows that controlled aging can significantly coarsen coherent κ′ carbides, achieving yield strengths exceeding 1.2 GPa primarily through precipitation strengthening. Ji et al. [86] elucidated the sequential evolution of short-range ordered clusters, nano-sized intragranular κ-carbides, and coarse intergranular κ-carbides in strip-cast steels, noting that only the coarse intergranular precipitates severely compromise material ductility and toughness. For medium-Mn systems, Liu et al. [85] reported that during prolonged aging, κ-carbides may grow via a γ→α+κ eutectoid reaction, forming a lamellar structure that enhances strength but significantly reduces elongation. Brasche et al. [91] further indicated that pre-deformation alters the precipitation pathway of κ-carbides, as dislocations act as heterogeneous nucleation sites and interact with recovery/recrystallization processes, thereby tuning the strength-ductility balance. Li et al. [92] proposed a novel strategy involving the cooperative precipitation of the α-phase and κ-carbides, which can simultaneously enhance shape memory functionality and mechanical performance, offering a new paradigm for alloy design.

## 5. Summary

This work reviews current progress on κ-phase precipitation behavior in Fe-Mn-Al-C austenitic lightweight steels, emphasizing the precipitation types, kinetics, compositional, and process influencing factors. The main conclusions are summarized as follows:

1. Structure and morphology of k-phase: The κ-phase exhibits an ordered L1_2_-crystal structure with C atoms preferentially occupying body-centered positions in the austenitic unit cell. Two morphologically distinct precipitation modes have been identified: intragranular κ′-carbides that nucleate homogeneously within austenite grains and intergranular κ*-carbides that preferentially form at grain boundaries. These morphological variants exert contrasting effects on mechanical behavior. Intergranular κ*-carbides promote grain boundary embrittlement by disrupting interfacial cohesion, leading to substantial degradation in ductility and fracture toughness—rendering them detrimental to structural integrity. In contrast, finely dispersed intragranular κ′-carbides with nanoscale dimensions contribute beneficially to mechanical properties. During plastic deformation, multiple concurrent deformation mechanisms, including Dynamic Slip Band Refinement (DSBR), Shear Band-Induced Plasticity (SIP), and Microband-Induced Plasticity (MBIP), activate. The synergistic operation of these mechanisms enhances work hardening capacity while maintaining good ductility, thereby improving the strength-ductility balance.
2. Alloying elements exert distinct influences on κ-carbide precipitation behavior through their effect on thermodynamic driving forces and diffusion kinetics. Mn moderately retards k-phase formation by stabilizing the austenite matrix and reducing C activity, whereas Al and C strongly promote precipitation by increasing the chemical driving force for the ordered phase formation. Quantitative metallographic analysis establishes critical precipitation thresholds: intragranular κ′-carbides require >6.2 wt.% Al and >1.0 wt.% C, whereas intergranular κ*-carbides initiate at >5.5 wt.% Al and >0.7 wt.% C. The additional influences of Cr, Ni, Cu, V, etc., on the driving force for k-phase or other secondary phase precipitation have also been revealed.
3. Thermal processing strategies that leverage the differential precipitation kinetics of κ′ and κ* variants have been discussed. Accelerated cooling from the hot-rolling or solution treatment temperature is proposed to minimize intergranular k*; controlled aging treatment (500–700 °C) at optimized time is proposed to control the size and volume fraction of intragranular κ′-carbide precipitation, while avoiding precipitate coarsening or detrimental κ*-phase formation.
4. Based on the significant progress in understanding κ-carbide precipitation behavior, future work to facilitate industrial production of these advanced austenitic steels is perspective, including leveraging computational approaches including machine learning, CALPHAD-based high-throughput calculations, and multi-objective optimization to accelerate alloy design for the complex Fe-Mn-Al-C-X systems; scaling the laboratory processing protocols to industrial production, particularly the controlled rolling- heat treatment; exploring their in-service performance especially high/low temperature mechanical properties, fatigue resistance as well as environmental fracture behavior.

## Data Availability

No new data were created or analyzed in this study. Data sharing is not applicable to this article.

## References

[1] Chen S., Rana R., Haldar A., Ray R.K. (2017). Current state of Fe-Mn-Al-C low density steels. Prog. Mater. Sci..

[2] Kies F., Wu X., Hallstedt B., Li Z., Haase C. (2021). Enhanced precipitation strengthening of multi-principal element alloys by κ- and B2-phases. Mater. Des..

[3] Liu L., Li C., Yang Y., Luo Z., Song C., Zhai Q. (2017). A simple method to produce austenite-based low-density Fe–20Mn–9Al–0.75C steel by a near-rapid solidification process. Mater. Sci. Eng. A.

[4] Li J., Dong X., Wang H., Deng X. (2025). Synergistic Enhancement of Strength and Ductility in Fe-20Mn-9Al-1.2C-2Ni Lightweight Steel via Intergranular Precipitate Dissolution and Grain Boundary Engineering. Steel Res. Int..

[5] Rahnama A., Kotadia H., Clark S., Janik V., Sridhar S. (2018). Nano-mechanical properties of Fe-Mn-Al-C lightweight steels. Sci. Rep..

[6] Wang J., Yang M., Wang W., Wu X., Yuan F. (2025). Optimization of size of coherent κ’–nanocarbides for achieving superior abrasion and wear resistance in a lightweight steel. Mater. Charact..

[7] Mapelli C., Villa G., Barella S., Gruttadauria A., Mombelli D., Veys X., Duprez L. (2021). JMAK model applied on the κ-carbide precipitation in FeMnAlC steels. J. Mater. Res. Technol..

[8] Xiong Y., Guo X., Dong H. (2024). Impact of Size and Distribution of k-Carbides on the Hydrogen Embrittlement and Trapping Behaviors of a Fe-Mn-Al-C Low-Density Steel. Materials.

[9] Kimura Y., Handa K., Hayashi K., Mishima Y. (2004). Microstructure control and ductility improvement of the two-phase γ-Fe/κ-(Fe, Mn)3AlC alloys in the Fe–Mn–Al–C quaternary system. Intermetallics.

[10] Kim H., Spivack A.J., Menden-Deuer S. (2013). pH alters the swimming behaviors of the raphidophyte Heterosigma akashiwo: Implications for bloom formation in an acidified ocean. Harmful Algae.

[11] Rana R. (2014). Low-Density Steels. JOM.

[12] Frommeyer G., Brüx U. (2006). Microstructures and mechanical properties of high-strength Fe–Mn–Al–C light-weight triplex steels. Steel Res. Int..

[13] Drouven C., Hallstedt B., Song W., Bleck W. (2019). Experimental observation of κ-phase formation sequences by in-situ synchrotron diffraction. Mater. Lett..

[14] Tuan Y.H., Lin C.L., Chao C.G., Liu T.F. (2008). Grain Boundary Precipitation in Fe-30Mn-9Al-5Cr-0.7C Alloy. Mater. Trans..

[15] Chao C.Y., Hwang C.N., Liu T.F. (1996). Grain boundary precipitation in an Fe-7.8Al-31.7Mn-0.54C alloy. Scr. Metall. Mater..

[16] Chao C.Y., Hwang C.N., Liu T.F. (1996). Grain boundary precipitation behaviours in an Fe-9.8Al-28.6Mn-0.8Si-1.0C alloy. Scr. Mater..

[17] Witkowska M., Chronowska-Przywara K., Kowalska J., Zielinska-Lipiec A. (2024). Microstructure and Texture Evolution of X85MnAl29-9 Steel During Aging. Materials.

[18] Cheng W.-C. (2014). Phase Transformations of an Fe-0.85 C-17.9 Mn-7.1 Al Austenitic Steel After Quenching and Annealing. JOM.

[19] Zhang B.-G., Zhang X.-M., Liu H.-T. (2021). Precipitation behavior of B2 and κ-carbide during aging and its effect on mechanical properties in Al-containing high strength steel. Mater. Charact..

[20] Rahnama A., Dashwood R., Sridhar S. (2017). A phase-field method coupled with CALPHAD for the simulation of ordered κ-carbide precipitates in both disordered γ and α phases in low density steel. Comput. Mater. Sci..

[21] Yao M.J., Welsch E., Ponge D., Haghighat S.M.H., Sandlöbes S., Choi P., Herbig M., Bleskov I., Hickel T., Lipinska-Chwalek M. (2017). Strengthening and strain hardening mechanisms in a precipitation-hardened high-Mn lightweight steel. Acta Mater..

[22] Sato K., Tagawa K., Inoue Y. (1989). Spinodal Decomposition and Mechanical Properties of an Austenitic Fe-30wt.%Mn-9wt.%AI-0.9wt.%C Alloy. Mater. Sci. Eng. A.

[23] Sato K., Tagawa K., Inoue Y. (1990). Modulated Properties Structure and Magnetic of Age-Hardenable Fe-Mn-AI-C Alloys. Metall. Trans. A.

[24] Bartlett L.N., Van Aken D.C., Medvedeva J., Isheim D., Medvedeva N.I., Song K. (2014). An Atom Probe Study of Kappa Carbide Precipitation and the Effect of Silicon Addition. Metall. Mater. Trans. A.

[25] Cheng W.-C., Cheng C.-Y., Hsu C.-W., Laughlin D.E. (2015). Phase transformation of the L1 2 phase to kappa-carbide after spinodal decomposition and ordering in an Fe–C–Mn–Al austenitic steel. Mater. Sci. Eng. A.

[26] Lu W.J., Qin R.S. (2016). Influence of κ-carbide interface structure on the formability of lightweight steels. Mater. Des..

[27] Zhang J., Jiang Y., Zheng W., Liu Y., Addad A., Ji G., Song C., Zhai Q. (2021). Revisiting the formation mechanism of intragranular κ-carbide in austenite of a Fe-Mn-Al-Cr-C low-density steel. Scr. Mater..

[28] Chu S.M., Kao P.W., Gan D. (1992). Growth Kinetics of κ-Carbide Particles in Fe-30Mn-10AI-1C-1Si Alloy. Scr. Metall. Mater..

[29] Emo J., Maugis P. (2017). Atomic mean-field model of E21 ordering in γ-iron-aluminium-carbon alloys. J. Alloys Compd..

[30] Zhang T., Wei H., Zhang K., Fu X., Cao Y., Zhang X., Li Z., Liu H. (2023). Effect of cooling medium on the κ carbide precipitation behavior, microstructure and impact properties of FeMnAlC low-density steel. Mater. Today Commun..

[31] Park K.-T., Jin K.G., Han S.H., Hwang S.W., Choi K., Lee C.S. (2010). Stacking fault energy and plastic deformation of fully austenitic high manganese steels: Effect of Al addition. Mater. Sci. Eng. A.

[32] Yoo J.D., Park K.-T. (2008). Microband-induced plasticity in a high Mn–Al–C light steel. Mater. Sci. Eng. A.

[33] Yoo J.D., Hwang S.W., Park K.T. (2009). Origin of Extended Tensile Ductility of a Fe-28Mn-10Al-1C Steel. Metall. Mater. Trans. A.

[34] Park K.-T. (2013). Tensile deformation of low-density Fe–Mn–Al–C austenitic steels at ambient temperature. Scr. Mater..

[35] Choi K., Seo C.-H., Lee H., Kim S.K., Kwak J.H., Chin K.G., Park K.-T., Kim N.J. (2010). Effect of aging on the microstructure and deformation behavior of austenite base lightweight Fe–28Mn–9Al–0.8C steel. Scr. Mater..

[36] Chang K.M., Chao C.G., Liu T.F. (2010). Excellent combination of strength and ductility in an Fe–9Al–28Mn–1.8C alloy. Scr. Mater..

[37] Lin C.L., Chao C.G., Bor H.Y., Liu T.F. (2010). Relationship between Microstructures and Tensile Properties of an Fe-30Mn-8.5Al-2.0C Alloy. Mater. Trans..

[38] Lin C.-L., Chao C.-G., Juang J.-Y., Yang J.-M., Liu T.-F. (2014). Deformation mechanisms in ultrahigh-strength and high-ductility nanostructured FeMnAlC alloy. J. Alloys Compd..

[39] Gutierrez-Urrutia I., Raabe D. (2012). Multistage strain hardening through dislocation substructure and twinning in a high strength and ductile weight-reduced Fe–Mn–Al–C steel. Acta Mater..

[40] Gutierrez-Urrutia I., Raabe D. (2014). High strength and ductile low density austenitic FeMnAlC steels: Simplex and alloys strengthened by nanoscale ordered carbides. Mater. Sci. Technol..

[41] Springer H., Raabe D. (2012). Rapid alloy prototyping: Compositional and thermo-mechanical high throughput bulk combinatorial design of structural materials based on the example of 30Mn–1.2C–xAl triplex steels. Acta Mater..

[42] Gutierrez-Urrutia I., Raabe D. (2011). Dislocation and twin substructure evolution during strain hardening of an Fe–22wt.% Mn–0.6wt.% C TWIP steel observed by electron channeling contrast imaging. Acta Mater..

[43] Gutierrez-Urrutia I., Raabe D. (2013). Influence of Al content and precipitation state on the mechanical behavior of austenitic high-Mn low-density steels. Scr. Mater..

[44] Welsch E., Ponge D., Hafez Haghighat S.M., Sandlöbes S., Choi P., Herbig M., Zaefferer S., Raabe D. (2016). Strain hardening by dynamic slip band refinement in a high-Mn lightweight steel. Acta Mater..

[45] Frommeyer G., Brüx U., Neumann P. (2003). Supra-Ductile and High-Strength Manganese-TRIP/TWIP Steels for High Energy Absorption Purposes. ISIJ Int..

[46] Lai H.J., Wan C.M. (1989). The study of work hardening in Fe-Mn-AI-C alloys. J. Mater. Sci..

[47] Frommeyer G., Drewes E.J., Engl B. (2000). Physical and mechanical properties of iron-aluminium(Mn, Si) lightweight steels. Rev. Met. Paris.

[48] Song W., Zhang W., von Appen J., Dronskowski R., Bleck W. (2015). κ-Phase Formation in Fe-Mn-Al-C Austenitic Steels. Steel Res. Int..

[49] Saeed-Akbari A., Imlau J., Prahl U., Bleck W. (2009). Derivation and Variation in Composition-Dependent Stacking Fault Energy Maps Based on Subregular Solution Model in High-Manganese Steels. Metall. Mater. Trans. A.

[50] Song W., Ingendahl T., Bleck W. (2014). Control of Strain Hardening Behavior in High-Mn Austenitic Steels. Acta Metall. Sin..

[51] Kim C., Terner M., Hong H.-U., Lee C.-H., Park S.-J., Moon J. (2020). Influence of inter/intra-granular κ-carbides on the deformation mechanism in lightweight Fe-20Mn-11.5Al-1.2C steel. Mater. Charact..

[52] Liu D., Ding H., Han D., Cai M. (2023). Effect of grain interior and grain boundary κ-carbides on the strain hardening behavior of medium-Mn lightweight steels. Mater. Sci. Eng. A.

[53] Ding H., Liu D., Cai M., Zhang Y. (2022). Austenite-Based Fe-Mn-Al-C Lightweight Steels: Research and Prospective. Metals.

[54] Han D., Ding H., Liu D., Rolfe B., Beladi H. (2020). Influence of C content and annealing temperature on the microstructures and tensile properties of Fe–13Mn–8Al–(0.7, 1.2)C steels. Mater. Sci. Eng. A.

[55] Liu D., Ding H., Hu X., Han D., Cai M. (2020). Dynamic recrystallization and precipitation behaviors during hot deformation of a κ-carbide-bearing multiphase Fe–11Mn–10Al–0.9C lightweight steel. Mater. Sci. Eng. A.

[56] García-Domínguez M., Mejía I., Schell N., Stark A., Cabrera J.M., Barriobero-Vila P. (2025). Evolution of precipitation in a duplex Fe-Mn-Al-C low-density steel revealed by in situ high-energy synchrotron X-ray diffraction. Mater. Today Commun..

[57] Liu D., Tong Z., Han D., Ding H., Cai M., Zhao K., Li H., Niu S. (2024). Study on the influence of κ-carbide on the high temperature flow behavior of the medium-Mn lightweight steel: Modeling and characterization. Mater. Sci. Eng. A.

[58] Li S., Li D., Lu H., Cao P., Xie R. (2022). Effect of κ Carbides on Deformation Behavior of Fe-27Mn-10Al-1C Low Density Steel. Crystals.

[59] Zhao C., Song R., Zhang L., Yang F., Kang T. (2016). Effect of annealing temperature on the microstructure and tensile properties of Fe–10Mn–10Al–0.7C low-density steel. Mater. Des..

[60] Tang Y., Ji P., Li B., Chen B., Shi H., Guo Y., Zhang S., Zhang J., Zhang X., Liu R. (2024). Tribology, corrosion, and tribocorrosion performance of aged lightweight steels: Effects of oxide film and carbide. Corros. Sci..

[61] Liu J., Li L., Yang S., Ding C., Wang E., Yu X., Wu H., Niu G. (2023). Effect of intragranular κ carbides and intergranular precipitates on the hot deformation mechanism and dynamic recrystallization mechanism of Fe–28Mn–11Al–1.5C–5Cr lightweight steel. J. Mater. Res. Technol..

[62] Zhu H., Gao Q., Tang Y., Zou Y., Ding H. (2025). Achieving a significant enhancement of strength in Fe-24Mn-8Al-1C austenitic lightweight steels by aging treatment. Mater. Charact..

[63] Zhou Y., Xiao L., Li Y., Deng X., Wang Z. (2025). Tailoring yield strength in Fe-20Mn-9Al-1.5C-3Cr-2Ni austenitic lightweight steels achieved by larger volume and size κ′-Carbide under extended aging. Mater. Lett..

[64] An Y.F., Chen X.P., Mei L., Ren P., Wei D., Cao W.Q. (2024). Precipitation transformation pathway and mechanical behavior of nanoprecipitation strengthened Fe–Mn–Al–C–Ni austenitic low-density steel. J. Mater. Sci. Technol..

[65] Moon J., Park S.-J., Jang J.H., Lee T.-H., Lee C.-H., Hong H.-U., Han H.N., Lee J., Lee B.H., Lee C. (2018). Investigations of the microstructure evolution and tensile deformation behavior of austenitic Fe-Mn-Al-C lightweight steels and the effect of Mo addition. Acta Mater..

[66] Wang Y., Hu F., Wang Z., Fu K., Li W., Wang J., Guo J. (2022). Microstructure and Constitutive Equation of Hot Compressive Fe-15Mn-15Al-5Ni-1C Low-Density Steel. Materials.

[67] Ma T., Gao J., Li H., Li C., Zhang H., Li Y. (2021). Microband-Induced Plasticity in a Nb Content Fe–28Mn–10Al–C Low Density Steel. Metals.

[68] Ren P., Chen X.P., Wang C.Y., Zhou Y.X., Cao W.Q., Liu Q. (2021). Evolution of microstructure, texture and mechanical properties of Fe–30Mn–11Al–1.2C low-density steel during cold rolling. Mater. Charact..

[69] Kim C., Hong H.-U., Jang J.H., Lee B.H., Park S.-J., Moon J., Lee C.-H. (2021). Reverse partitioning of Al from κ-carbide to the γ-matrix upon Ni addition and its strengthening effect in Fe–Mn–Al–C lightweight steel. Mater. Sci. Eng. A.

[70] Park S.-W., Park J.Y., Cho K.M., Jang J.H., Park S.-J., Moon J., Lee T.-H., Shin J.-H. (2018). Effect of Mn and C on Age Hardening of Fe–Mn–Al–C Lightweight Steels. Met. Mater. Int..

[71] Fartushna I., Bajenova I., Khvan A., Cheverikin V., Ivanov D., Shilundeni S., Alpatov A., Sachin K., Hallstedt B. (2018). Experimental investigation of solidification and isothermal sections at 1000 and 1100 ℃ in the Al-Fe-Mn-C system with special attention to the kappa-phase. J. Alloys Compd..

[72] Ding H., Han D., Zhang J., Cai Z., Wu Z., Cai M. (2016). Tensile deformation behavior analysis of low density Fe–18Mn–10Al–xC steels. Mater. Sci. Eng. A.

[73] Huang H., Gan D., Kao P.W. (1994). Effect of alloying additions on the j phase precipitation in austenitic Fe-Mn-Al-C alloys. Scr. Metall. Mater..

[74] Peng T., Yu H., Huang J., Fang W., Li C., Yao Z., Zhang X., Feng J., Ji P., Xia C. (2024). Deciphering the composition-microstructure correlation in low-density FeMnAlC steels with machine learning. Comput. Mater. Sci..

[75] Ishida K., Ohtanl H., Satoh N., Kainuma R., Nishizaw T. (1990). Phase equilibria in Fe-Mn-Al-C alloys. ISIJ Int..

[76] Chin K.-G., Lee H.-J., Kwak J.-H., Kang J.-Y., Lee B.-J. (2010). Thermodynamic calculation on the stability of (Fe,Mn)3AlC carbide in high aluminum steels. J. Alloys Compd..

[77] Bale C.W., Bélisle E., Chartrand P., Decterov S.A., Eriksson G., Hack K., Jung I.H., Kang Y.B., Melançon J., Pelton A.D. (2009). FactSage thermochemical software and databases—Recent developments. Calphad.

[78] Wang Z., Lu W., Zhao H., He J., Wang K., Zhou B., Ponge D., Raabe D., Li Z. (2020). Formation mechanism of κ-carbides and deformation behavior in Si-alloyed FeMnAlC lightweight steels. Acta Mater..

[79] Moon J., Park S.-J., Jang J.H., Lee T.-H., Lee C.-H., Hong H.-U., Suh D.-W., Kim S.H., Han H.N., Lee B.H. (2017). Atomistic investigations of κ-carbide precipitation in austenitic Fe-Mn-Al-C lightweight steels and the effect of Mo addition. Scr. Mater..

[80] Moon J., Ha H.-Y., Park S.-J., Lee T.-H., Jang J.H., Lee C.-H., Han H.N., Hong H.-U. (2019). Effect of Mo and Cr additions on the microstructure, mechanical properties and pitting corrosion resistance of austenitic Fe-30Mn-10.5Al-1.1C lightweight steels. J. Alloys Compd..

[81] Moon J., Park S.-J., Kim S.-D., Jang J.H., Lee T.-H., Lee C.-H., Lee B.H., Hong H.-U., Han H.N. (2019). Phase transformation mechanism and hardness during ageing of an austenitic Fe-30Mn-10.5Al-1.1C-3Mo lightweight steel. J. Alloys Compd..

[82] Bai R., Du Y., He X., Zhang Y. (2024). The Influence of Cr Addition on the Microstructure and Mechanical Properties of Fe-25Mn-10Al-1.2C Lightweight Steel. Metals.

[83] Xie Z., Hui W., Bai S., Zhang Y., Zhao X., Li B. (2023). Effects of annealing temperature and V addition on microstructure and mechanical properties of Fe-Mn-Al-C austenitic low-density steel. Mater. Today Commun..

[84] Yang L., Li Z., Li X., Zhang Y., Han K., Song C., Zhai Q. (2020). An Enhanced Fe–28Mn–9Al–0.8C Lightweight Steel by Coprecipitation of Nanoscale Cu-Rich and κ-Carbide Particles. Steel Res. Int..

[85] Liu D., Ding H., Han D., Cai M., Lee Y.-K. (2020). Microstructural evolution and tensile properties of Fe–11Mn–10Al-1.2C medium-Mn lightweight steel. Mater. Sci. Eng. A.

[86] Ji F., Li C., Song W., Bleck W., Wang G. (2024). Effect of Second Phase on the Tensile Properties of a High-Mn High-Al Austenitic Lightweight Steel Processed by Thin-Strip Casting. Steel Res. Int..

[87] Feng Y., Song R., Pei Z., Song R., Dou G. (2018). Effect of Aging Isothermal Time on the Microstructure and Room-Temperature Impact Toughness of Fe–24.8Mn–7.3Al–1.2C Austenitic Steel with κ-Carbides Precipitation. Met. Mater. Int..

[88] Pang J.C., Yang W.F., Wang G.D., Zheng S.J., Misra R.D.K., Yi H.L. (2022). Divorced eutectoid transformation in high-Al added steels due to heterogenous nucleation of κ-carbide. Scr. Mater..

[89] Zhou J., Zhang J., Liu M., Ma Z., Yang Y., Xu Z., Song C. (2022). Relationship between Austenite-Stabilizing Elements and Austenite Fraction in Near-Rapidly Solidified Fe–Mn–Al–C Lightweight Steel. Steel Res. Int..

[90] Lopez-Hirata V.M., Perez-Badillo E., Saucedo-Muñoz M.L., Hernandez-Santiago F., Villegas-Cardenas J.D. (2024). Phase Transformations after Heat Treating an As-Cast Fe-30Mn-8.8Al-0.3Si-0.15C Steel. Metals.

[91] Brasche F., Haase C., Lipińska-Chwałek M., Mayer J., Molodov D.A. (2020). Combined κ-carbide precipitation and recovery enables ultra-high strength and ductility in light-weight steels. Mater. Sci. Eng. A.

[92] Li S., Huang Y., Cai L., Peng H., Yan J., Wen Y. (2023). Simultaneously improving memory effect and mechanical properties in Cu-based alloys by α phase spheroidization and Fe alloying: A CuAlMnFe as an example. Mater. Sci. Eng. A.

